# Supplementary material for: Making an economic argument for investment in global mental health: The case of conflict-affected refugees and displaced people
Source: Glob Ment Health (Camb). 2023 Mar 2;10:e10. doi: 10.1017/gmh.2023.1 (PMC10579650; doi:10.1017/gmh.2023.1)
Supplement: Supplementary file 1 [file S2054425123000018sup001.docx]

**Supplementary File: Scoping Review Search Strategy**

**PubMed**

1. Costs and Costs Analysis/
2. Mental Health/
3. Mental Disorders/
4. Trauma and Stressor Related Disorders/
5. Psychological Stress/
6. Warfare and Armed Conflicts/
7. Refugees/
8. Conflict.ti
9. War.ti
10. 8 OR 9
11. 2-5/OR
12. 1 AND 7 AND 11
13. 1 AND 10 AND 11
14. 1 AND 7
15. 12-14/OR

**Psychinfo**

1. Costs and Costs Analysis/
2. Mental Health/
3. Mental Disorders/
4. (Trauma OR “Stressor Related”).ti, ab
5. Psychological Stress/
6. Warfare.ti,ab
7. Conflict.ti,ab
8. Refugees/
9. 6 OR 7
10. 2-5/OR
11. 1 AND 8 AND 10
12. 1 AND 9 AND 10
13. 1 AND 8
14. 11-13/OR

**EMBASE**

1. Economic evaluation/
2. Mental Health/
3. Mental Disease/
4. Post Traumatic Stress Disorder/
5. War/
6. Forced migrant/
7. Warfare.ti,ab
8. Conflict.ti,ab
9. 8 OR 9
10. 2-5/OR
11. 1 AND 7
12. 1 AND 10 AND 11
13. 1 AND 6 AND 11
14. 12-14/OR

**Global Health**

1. Economic analysis/
2. Mental Health/
3. Mental Disorders/
4. Post Traumatic Stress Disorder/
5. War/
6. Refugees/
7. Migrants/
8. 2-4/OR
9. 6-7/OR
10. 1 AND 8 AND 9
11. 1 AND 6
12. 10-11/OR

**LILACS/DESASTRES/IBECS/PAHO,**

1. Cost.ti, ab
2. Mental.ti, ab
3. Desplazamiento OR Refugiados OR Displaced OR Refugees OR Migrantes OR Migrants
4. Main Subject: Mental Health
5. Main Subject: Refugees
6. 4-5/OR
7. Type of Study: Evaluation Studies
8. Type of Study: Health Economic Evaluation
9. Type of Study: Programme Evaluation
10. Type of Study: Disaster Planning
11. 7-10/OR
12. 1 AND 3 AND 4
13. 1 AND 6 AND 11
14. 12-13/OR
